# Influencing Factors on the Overestimation of Self-Reported Physical Activity: A Cross-Sectional Analysis of Low Back Pain Patients and Healthy Controls

**DOI:** 10.1155/2016/1497213

**Published:** 2016-05-19

**Authors:** Andrea Schaller, Kevin Rudolf, Lea Dejonghe, Christopher Grieben, Ingo Froboese

**Affiliations:** ^1^IST University of Applied Sciences, Erkrather Straße 220 a-c, 40233 Düsseldorf, Germany; ^2^Institute of Health Promotion and Clinical Movement Science, German Sport University Cologne, Am Sportpark Muengersdorf 6, 50933 Cologne, Germany; ^3^Center for Health and Physical Activity, German Sport University Cologne, Am Sportpark Muengersdorf 6, 50933 Cologne, Germany

## Abstract

*Introduction.* The aim of the present study was to determine the closeness of agreement between a self-reported and an objective measure of physical activity in low back pain patients and healthy controls. Beyond, influencing factors on overestimation were identified.* Methods.* 27 low back pain patients and 53 healthy controls wore an accelerometer (objective measure) for seven consecutive days and answered a questionnaire on physical activity (self-report) over the same period of time. Differences between self-reported and objective data were tested by Wilcoxon test. Bland-Altman analysis was conducted for describing the closeness of agreement. Linear regression models were calculated to identify the influence of age, sex, and body mass index on the overestimation by self-report.* Results.* Participants overestimated self-reported moderate activity in average by 42 min/day (*p* = 0.003) and vigorous activity by 39 min/day (*p* < 0.001). Self-reported sedentary time was underestimated by 122 min/day (*p* < 0.001). No individual-related variables influenced the overestimation of physical activity. Low back pain patients were more likely to underestimate sedentary time compared to healthy controls.* Discussion.* In rehabilitation and health promotion, the application-oriented measurement of physical activity remains a challenge. The present results contradict other studies that had identified an influence of age, sex, and body mass index on the overestimation of physical activity.

## 1. Introduction

Lack of physical activity is a primary contributor to many chronic diseases [1–3] and evidence on the positive effects of physical activity on health is proven [4]. As such, the importance of promoting physical activity is a major field in rehabilitation and health promotion and has created a need for precise, economic, and practicable tools for measuring the physical activity level of a person [5].

**Figure 1:**
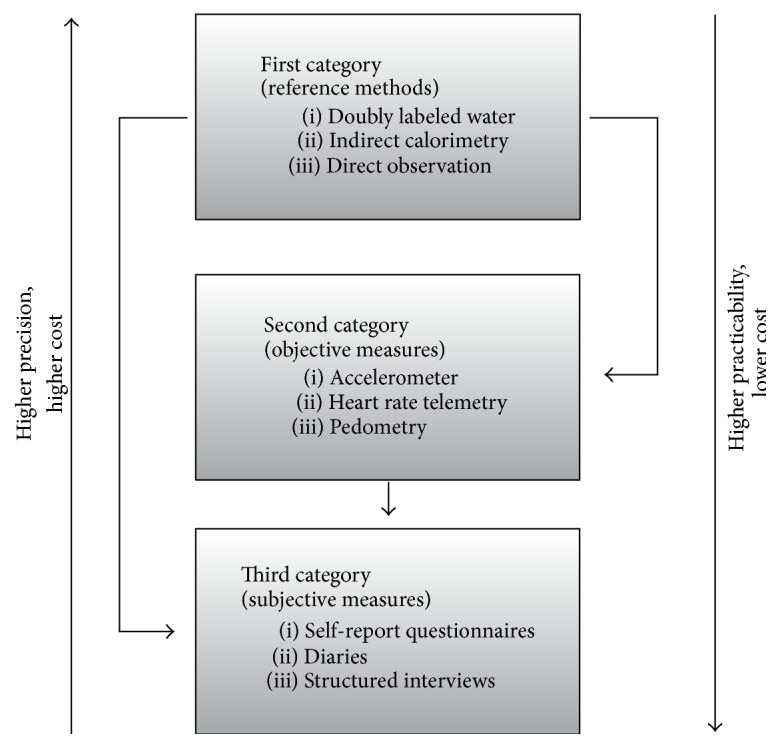
Measurement of physical activity (modified according to Müller et al. [8] and Beneke and Leithäuser [9]).

Assessing physical activity is very complex due to several aspects. First, physical activity encompasses any bodily movement produced by skeletal muscles that requires energy expenditure and therefore is not related to sports only [6]. The increasingly recognized lifestyle physical activities especially in daily routine like workplace and leisure time activities or active mobility, respectively, are difficult to assess and hardly fully observable [7]. Second, the instruments applied, on the one hand, are supposed to show valid and reliable results and, on the other hand, need to be practicable to ensure compliance of the participants. The available instruments for measuring physical activity can be distinguished in three categories according to their precision and practicability [8, 9]: reference methods, objective measures, and subjective measures (see Figure 1).

*Reference methods,* for example, direct observation and indirect calorimetry, are of high precision and high cost;* self-report* instruments (e.g., questionnaires, physical activity diaries) are considered to be practicable and low in cost but rather imprecise. Between these extremes,* objective measures* like accelerometers or pedometers are appraised for medium precision and cost as well as medium practicability.

While reference methods are mainly applied in basic research, objective instruments, especially accelerometers, are an accepted measurement tool in application-oriented studies. Nevertheless, the use of accelerometers still requires a high extent of participant cooperation and compliance. Besides, accelerometers often are too expensive to be widely used in physical activity studies [7]. In consequence, assessing physical activity by self-report questionnaire is of high relevance not only in surveys but also in application-oriented studies.

Several studies showed that self-reported physical activity tends to be overestimated compared to objective measure but, up to now, no clear trends of overestimation could be identified [10, 11]. Regarding sedentary time, studies showed a tendency for underestimation by self-report measures [12–14]. In this regard, low back pain patients seem to have even more problems estimating their physical activity levels than healthy controls [15].

Besides the scientific perspective, the misperception of self-reported physical activity is an important aspect in the practice of rehabilitation and health promotion. Misperception, especially the overestimation of one's own physical activity, is described as a potential barrier of behavior change [16]. In consequence, the question arises regarding what factors are associated with the agreement of subjective and objective physical activity.

The present paper aims to explore the closeness of agreement of self-reported and objective physical activity (question 1) and explores influencing factors on the overestimation of moderate and vigorous physical activity (question 2) as well as influencing factors on the underestimation of sedentary time (question 3).

## 2. Material and Methods

The present study was an additional substudy within the scope of two different main studies on physical activity promotion. In brief, both studies, the* Movement Coaching* study and the* Make Move* study, aimed at target-group specific physical activity promotion. While the main study of* Movement Coaching* (October 2012 to September 2015) focused on persons undergoing rehabilitation because of low back pain, the main study of* Make Move* focused on healthy vocational students (September 2013 to December 2014).

The* Movement Coaching* study and the* Make Move* study were conducted in compliance with the Helsinki Declaration and were approved by the Ethics Committee of the German Sport University Cologne, each.

*Movement Coaching* is registered in the German Clinical Trials Register (DRKS00004878) and the study protocol has already been described elsewhere [17]. For further information on* Make Move*, see Frick et al. [18].

### 2.1. Sample

For the present evaluation, participants were recruited from the two different main studies (*Movement Coaching* and* Make Move*). Eligible participants from each study were invited to participate in our associated additional study on the association of self-reported and objective physical activity. During an information seminar, we informed the participants that we investigated the physical activity behavior during daily routine.

Inclusion criteria were (1) being a participant of the* Movement Coaching* or the* Make Move* study, (2) distance to the study site in Cologne, Germany, <100 km, and (3) inpatient rehabilitation being completed (for participants from the* Movement Coaching* study).

Exclusion criteria were (1) limited mobility, (2) posttraumatic conditions within the last three months (e.g., an accident), (3) a surgery within the last three months, (4) age over 65 years, and (5) insufficient knowledge of the German language.

All participants provided informed consent. For the present evaluation, participants' data were collected in May and June 2014 (*Movement Coaching*) and from September to October 2013 (*Make Move*), respectively.

### 2.2. Study Design

The present study was conducted in a cross-sectional design. During the information seminar, the participants were instructed to wear an accelerometer for the next seven days and to complete a questionnaire on physical activity during the last week at the last day (seventh day). Participants were instructed to remove accelerometers for sleeping time and water activities only. All participants were indicated to continue their habitual daily routine.

### 2.3. Instruments Measuring Physical Activity

Self-reported physical activity was operationalized by the Global Physical Activity Questionnaire (GPAQ) [19, 20], which collects information on the intensity (moderate, vigorous) and the area of life (workplace, leisure time, and transportation) of physical activity during the last seven days as well as daily sedentary time. The GPAQ was originally designed to assess physical activity by interview but the comparability between self- and interviewer-administration modes of the GPAQ had been shown [21]. Compared with accelerometer data, the GPAQ provided low-to-moderate validity and generally acceptable evidence of reliability [22]. In comparison with the International Physical Activity Questionnaire, concurrent validity and reliability were moderate [20].

We evaluated self-reported minutes per week of total vigorous physical activity, minutes per week of total moderate physical activity, and sedentary time per day.

To obtain objective physical activity, a triaxial accelerometer (Actigraph GT3X+) was applied. Actigraph had been previously validated for adults against heart rate telemetry (*r* = 0.66–0.82) [23], indirect calorimetry (*r* = 0.66–0.88) [23, 24], and the doubly labeled water method (*r* = 0.26–0.58) [25]. Participants were instructed to wear the accelerometer on the right waist during waking hours for one week, removing them whilst showering, bathing, and swimming. Data were collected with a sample rate of 30 Hz and saved in 30-second epochs.

Before evaluation, the accelerometer data was processed using the Freedson and Troiano algorithms to obtain valid data on the duration and intensity of physical activity [26, 27]. According to the Freedson algorithm [26], intensity cut-points were set at 0–99 counts per minute (CPM) for sedentary behavior, 100–1951 CPM for light intensity, 1952–5724 CPM for moderate intensity, and >5725 CPM for vigorous intensity. Days with less than ten hours of recorded data were excluded from further evaluation.

For each participant, daily averages were calculated to get comparable units of objective data (daily average = (time of intensity specific activity)/(number of days with valid data)) and self-reported data on moderate and vigorous physical activity (daily average = (time of intensity specific activity)/7).

### 2.4. Further Variables

Besides self-reported and objective physical activity, the study group (healthy controls versus low back pain), sex (men versus women), age (years), and body mass index (kg/m^2^) were assessed. Furthermore, we assessed the objective physical activity status (active versus inactive). To differentiate active from physically inactive participants, objective accelerometer data were classified: according to the recommendations on health-enhancing physical activity [28], we defined participants showing ≥30 min moderate and/or vigorous physical activity/day as “active” and participants showing <30 min moderate and/or vigorous physical activity/day were defined as “inactive.”

### 2.5. Statistical Analysis

First, descriptive statistics (means, standard deviations, numbers, and percentages) were used to describe demographic characteristics and participants' objective and self-reported physical activity data. Differences between groups (healthy controls versus low back pain) were tested using Mann-Whitney *U* test and differences between self-reported and objective physical activity data (Actigraph versus GPAQ) were tested using the Wilcoxon signed-rank test.

The closeness of agreement between the two methods (question 1) was assessed using Bland-Altman plots [29], with the *x*-axis representing Actigraph data and the *y*-axis representing the difference between GPAQ and Actigraph (GPAQ-Actigraph) as value for the closeness of agreement of both methods. Additionally, participants' self-reports were classified as overestimation, underestimation, or realistic estimation when those values were higher than, lower than, or agreeing with the accelerometer data.

To identify independent variables associated with overestimation of moderate and vigorous physical activity, respectively, (question 2) as well as the underestimation of sedentary time (question 3), multiple linear regression models were calculated.

Overestimation of moderate physical activity was calculated by subtracting the objective moderate physical activity/day (measured by accelerometer) from the total moderate subjective moderate physical activity/day (measured by GPAQ). Overestimation of vigorous physical activity was calculated by subtracting the objective vigorous physical activity/day (measured by accelerometer) from the total vigorous subjective moderate physical activity/day (measured by GPAQ). Underestimation of sedentary time was calculated by subtracting the sedentary time/day (measured by accelerometer) from sedentary time/day measured by GPAQ.

To identify influencing factors on the overestimation of moderate and vigorous physical activity, respectively, the sample was restricted to participants that overestimated self-reported physical activity. To explore associated factors with the underestimation of sedentary time, the sample was restricted to participants that underestimated self-reported sedentary time.

Two different models were calculated. In the first model (model 1), sociodemographic and anthropometric variables (age, sex, and body mass index) and the study group (healthy controls versus low back pain) were included.

In model 2, self-reported workplace physical activity (min/week), self-reported leisure time physical activity (min/week), self-reported transportation activity (min/week), and objective achievement of the World Health Organization's recommendations on health-enhancing physical activity (≥30 min physical activity/day versus <30 min physical activity/day) were additionally included.

The principal assumptions of linear regression were tested. In all regression models, participants with missing values in dependent or independent variables were excluded.

For all statistical tests, significance level was set at *p* < 0.05. All analyses were run with IBM SPSS Statistics 22.

## 3. Results

**Figure 2:**
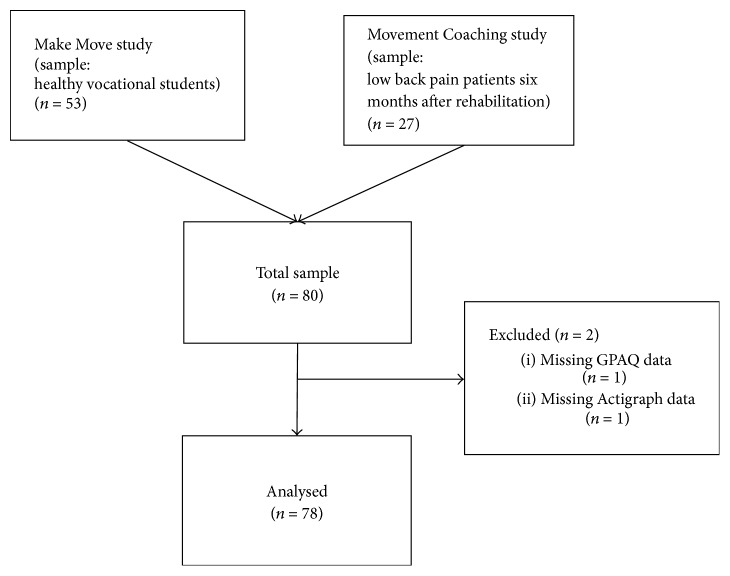
Flow-chart.

Informed consent was given by 80 participants who were assessed for eligibility. Two participants were excluded from the analysis due to incomplete self-reported or accelerometer data. 78 types of data were analysed.  Figure 2 shows the flow diagram illustrating the progress through the phases of the present study.

### 3.1. Sample Description

Detailed sample description of the* Make Move* study (healthy controls) can be found in Rudolf et al. (2015) [30].

The sample of the present evaluation included 53 healthy controls and 27 low back pain patients (see Table 1). Overall, 31 (40%) participants were female and the mean age was 30.7 (±15.3) years. The low back pain patients and healthy controls had statistically significant difference in age (*p* < 0.001) and body mass index (*p* < 0.001). In average, the healthy controls were significantly younger and showed a lower body mass index.

### 3.2. Descriptive Results on Subjective and Objective Physical Activity

Table 2 shows the descriptive results on objective and self-reported physical activity. The significance values on differences between the two groups as well as between the objective and self-reported physical activity data are presented.

In low back pain patients and healthy controls both, objective and self-reported daily sedentary time was much higher than objective and self-reported moderate and vigorous physical activity. In regard to self-reported sedentary time (healthy controls: 528 (±196); low back pain: 298 (±160); *p* < 0.001) and objective vigorous physical activity (healthy controls: 2 (±2); low back pain: 1 (±3); *p* = 0.030), statistically significant group differences between the healthy controls and the low back pain patients could be proved. Though the objective data indicated no group differences (healthy controls: 591 (±108); low back pain: 547 (±85); *p* = 0.153), low back pain patients (298 min/day) reported statistically significant lower sedentary time than healthy controls (528 min/day) (*p* < 0.001).

In healthy controls as well as low back pain patients, the objective and self-reported physical activity data were statistically significantly different in vigorous physical activity and sedentary time. Thereby, self-reported physical activity was significantly higher and self-reported sedentary time was statistically significantly lower than the accelerometer assessed data. Solely self-reported and objective data of moderate physical activity in healthy controls showed no difference (*p* = 0.153). Regarding the total sample, participants overestimated self-reported moderate activity (*p* = 0.003) and vigorous activity (*p* < 0.001). Self-reported sedentary time was significantly underestimated (*p* < 0.001) (Table 2).

### 3.3. Closeness of Agreement of Self-Reported and Objective Physical Activity

**Figure 3:**
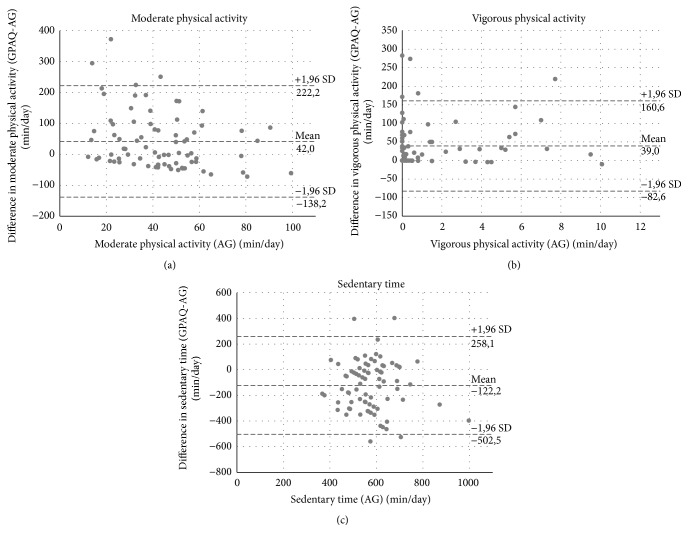
Bland-Altman plots for moderate physical activity, vigorous physical activity, and sedentary time. AG = Actigraph, GPAQ = Global Physical Activity Questionnaire; *n* = 78.

Figure 3 illustrates the closeness of agreement for the physical activity data for the total sample. In terms of moderate and vigorous physical activity as well as sedentary time, the plots showed a high range for the limit of agreement (see Figure 3) and statistically significant misperception (see Table 2).

Sedentary time showed the highest range for the limits of agreement (−503 min/day to 258 min/day) and therefore lower closeness of agreement between self-reported and objective data than moderate (−138 min/day to 222 min/day) and vigorous physical activity (−83 min/day to 161 min/day).

The Bland-Altman plots on the closeness agreement in moderate and vigorous physical activity showed a slight trend towards a closer agreement in higher objective physical activity as difference values get closer to zero (see Figure 3).

Table 3 shows the number of participants, over- and underestimating physical activity or sedentary time. While sedentary time was underestimated by most of the low back pain patients (*n* = 21; 84%) and the healthy controls (*n* = 35; 66%), moderate and vigorous physical activity were overestimated in most cases. Solely vigorous physical activity was estimated correctly by 15 participants (low back pain: *n* = 6; 24%; healthy controls: *n* = 9; 17%).

### 3.4. Influencing Factors on the Overestimation of Moderate and Vigorous Physical Activity

Neither the individual-related variables (age, sex, and body mass index) nor the study group (healthy controls versus low back pain) showed an association with the overestimation of moderate or vigorous physical activity in model 1 and model 2.

Explained variation of model 1 (moderate: *R* ^2^ = 0.069; vigorous: *R* ^2^ = 0.004) increased to 82% (moderate physical activity) and 78% (vigorous physical activity), when self-reported physical activity data in different areas of life were included (model 2). Thereby, higher self-perceived physical activity at the workplace and during leisure time led to an increase in overestimation of moderate (see Table 4) as well as vigorous physical activity (see Table 5).

### 3.5. Influencing Factors on the Underestimation of Sedentary Time

In line with the overestimation of moderate and vigorous physical activity, individual-related variables (age, sex, and body mass index) were not associated with the underestimation of sedentary time. However, an association with the study group could be proved (see Table 6).

Sedentary time was underestimated in both study groups (see Table 2) and the adjusted regression models 1 and 2 showed that low back pain patients underestimated sedentary time more than healthy controls.

In contrast to the overestimation of moderate and vigorous physical activity, self-perceived physical activity at the workplace and during leisure time did not contribute to a higher explanation of variance of the underestimation of sedentary time (see Table 6).

## 4. Discussion

The aim of this study was to explore the closeness of agreement between self-reported and objective physical activity. Associated factors with the overestimation of physical activity and the underestimation of sedentary time in low back pain patients and healthy controls were identified.

Our results showed that sedentary time was significantly underestimated and vigorous physical activity was significantly overestimated in both groups. Moderate physical activity was only overestimated in low back pain patients. The adjusted regression model showed that low back pain patients underestimated sedentary time more compared to healthy controls. No individual-related variables could be identified influencing the overestimation of physical activity.

Due to inconsistent definition of over- and underestimation in physical activity research, the comparison of the present results with other studies is limited. Our results showed worse awareness of sedentary time per day in low back pain patients. Also van Weering et al. (2011) pointed out that low back pain patients appeared to have more problems in estimating their activity levels compared to healthy controls. Hence, this study did not focus on sedentary time specifically [15].

Contrary to other studies that had been identifying an association of age [16, 31–33], sex [16, 32, 34], and body mass index [16, 32, 34, 35], the present results could not prove an influence of age, body mass index, and sex on the closeness of agreement between self-reported and objective physical activity.

As the anthropometric characteristics obtained in the present study did not contribute to explaining the overestimation of physical activity, the assumption arises that misperception of physical activity might mainly be due to the questionnaire used. By additionally including self-perceived physical activity in the regression model 2 and its related strong increase of the variance explanation, the present results underline the important challenge of measuring self-reported physical activity precisely [7, 36].

Self-assessment of physical activity depends on the accurate recall of the intensity, frequency, and duration of physical activity episodes [16]. In our study, participants filled out the GPAQ, an established questionnaire recommended from the World Health Organization [19]. The GPAQ assesses physical activity differentiated for the area of life (workplace, leisure time, and transportation) and the intensity (moderate, vigorous). For each area of life and each intensity, in a first question, the GPAQ asks dichotomously if physical activity is performed (yes/no). If the answer is yes, the subsequent second question asks for the number of days per week on which physical activity is performed (categorical; Likert scale from 1 to 7). Finally, the third question assesses the average hours and minutes per day performed (open question; interval scaled).

According to the present results, the GPAQ seems not to be valid in measuring the duration of physical activity as the degree of overestimation was associated with higher self-reported physical activity. Further research is needed to identify whether the GPAQ, or comparable questionnaires, might systematically overestimate physical activity by asking an open question on the duration of physical activity per day. This assumption is supported by Olsson et al. (2016) who point out that questions in the categorical mode provide stronger validity than open questions [5]. In consequence, questionnaires like the GPAQ might be appropriate to assess precisely if physical activity in a certain area of life is performed at all and how often per week.

Besides the construction of the questionnaire, it needs to be taken into account that the requirement of a realistic participant's self-perception of the duration and intensity of physical activity is a main challenge in the use of questionnaires [7]. Self-reported data especially from persons with low movement experience have to be interpreted very cautiously. Altschuler et al. (2009) indicate that assessing the intensity of physical activity by asking questions about an increase in breathing or pulse rate, as done in the GPAQ, offers a large margin of interpretation. In consequence, persons with low fitness level tend to overestimate the objective intensity of physical activity [37]. The present results tend to support this assumption. Though not significant, persons not achieving the World Health Organization's recommendation on health-enhancing physical activity tend to overestimate moderate physical activity compared to persons achieving WHO recommendations. Further studies should examine to what extent inactive persons and persons with low movement experience are able to differentiate between moderate and vigorous intensity of physical activity.

Though current self-report questionnaires may provide an approximation of physical activity at a population level, they may not be appropriate to assess precisely the intensity and duration of physical activity [38].

Also from a practical perspective, the awareness of one's own physical activity is an indispensable requirement regarding the individual's motivation to participate in a physical activity promotion program and realistic goal setting [39, 40]. The extent to how a person perceives his or her behavior is predictor of achieving behavioral change. So, the existence of a gap between what people think to do and what is actually done is an important aspect in rehabilitation as well as health promotion. Although it certainly is circular reasoning, whether the lack of precision of questionnaires measuring physical activity might be a consequence of the instrument's validity or the respondent's self-awareness, the present study supports the need for developing self-report instruments supporting self-awareness of physical activity.

The present study has several limitations. First, the merged sample of two different main studies needs to be mentioned. However, we cannot exclude study related effects like a self-selection bias. As participants in a physical activity intervention trial are likely to have had more interest in physical activity than nonparticipant, the results could show a higher closeness of agreement than in the general population.

It certainly would have been preferable to include more anthropometric and sociodemographic variables to explore the association with overestimation. This was not possible because of the integration of two different main studies. Besides, the cross-sectional design precludes the establishment of causality.

Another limitation remains regarding the small sample size of this study and the unequally numbered groups. In our opinion, it seems important to replicate the study with a larger group of participants.

Finally, the algorithms used for the processing of the Actigraph's raw data obviously influence the results. The use of other algorithms with lower cut-points for the classification of moderate and vigorous physical activity may result in smaller deviations and, hence, in differing results.

Despite the limitations mentioned, the present study has several strengths and therefore contributes substantially to the challenge of measuring physical activity in applied researches. As many studies already showed the lack of agreement between self-report and objective measure, the present study went beyond and focused on the degree of overestimation.

Moreover, the present evaluation asserts the degree of deviation by the Bland-Altman method whereas many studies only used a correlation coefficient to test the association between self-report and objective measures. The consideration of different target groups in physical activity promotion was another strength of the study as it gave us the opportunity to compare low back pain patients with healthy controls.

## 5. Conclusion

In rehabilitation and health promotion, the application-oriented measurement of physical activity remains a widely recognized challenge. The present results showed no association between overestimation and individual-related factors but stressed the need of further research on the development of self-report instruments to assess physical activity. The question arises regarding whether the participants have enough experience and knowledge to classify and quantify their individual physical activity correctly.

## Figures and Tables

**Table 1:** Sample description.

	Total sample (*n* = 78)	Healthy controls (*n* = 53)	Low back pain patients (*n* = 25)	*p*
*Sex*: female, *n* (%)	31 (40%)	23 (43%)	8 (32%)	0.337^1^
*Age* (years), mean (SD)	30.7 (±15.3)	20.7 (±3.1)	51.8 (±6.9)	**<0.001** ^2*∗*^
*Body mass index* (kg/m^2^), mean (SD)	25.8 (±5.2)	24.1 (±4.5)	29.4 (±4.9)	**<0.001** ^2*∗*^

^1^Chi-square test; ^2^Mann-Whitney *U* test; ^*∗*^statistically significant at the significance level *p* < 0.05.

**Table 2:** Descriptive results on objective and self-reported physical activity.

	Total sample (*n* = 78)	Healthy controls (*n* = 53)	Low back pain (*n* = 25)	*p* ^2^
Sedentary time (min/day)				
Objective physical activity (Actigraph) (mean (SD))	577 (±103)	591 (±108)	547 (±85)	0.153
Self-reported physical activity (GPAQ) (mean (SD))	454 (±214)	528 (±196)	298 (±160)	**<0.001** ^*∗*^
*p* ^1^	**<0.001** ^*∗*^	**0.009** ^*∗*^	**<0.001** ^*∗*^	
Moderate physical activity (min/day)				
Objective physical activity (Actigraph) (mean (SD))	43 (±19)	44 (±19)	41 (±20)	0.676
Self-reported physical activity (GPAQ) (mean (SD))	85 (±88)	69 (±75)	120 (±105)	0.050
*p* ^1^	**0.003** ^*∗*^	0.153	**0.004** ^*∗*^	
Vigorous physical activity (min/day)				
Objective physical activity (Actigraph) (mean (SD))	2 (±2)	2 (±2)	1 (±3)	**0.030** ^*∗*^
Self-reported physical activity (GPAQ) (mean (SD))	41 (±62)	38 (±54)	47 (±79)	0.706
*p* ^1^	**<0.001** ^*∗*^	**<0.001** ^*∗*^	**0.009** ^*∗*^	

^1^Wilcoxon signed-rank test for assessment differences; ^2^Mann-Whitney *U* test for group differences; ^*∗*^statistically significant at the significance level *p* < 0.05.

**Table 3:** Classification of over- and underestimation.

	Sedentary time (min/day) (*n* = 78)	Moderate physical activity (min/day) (*n* = 78)	Vigorous physical activity (min/day) (*n* = 78)
*Total sample*			
Overestimation of self-reported physical activity, *n* (%)	22 (28%)	41 (53%)	42 (54%)
Correct estimation of self-reported physical activity, *n* (%)	0	0	15 (19%)
Underestimation of self-reported physical activity, *n* (%)	56 (72%)	37 (47%)	21 (27%)

*Low back pain *			
Overestimation of self-reported physical activity, *n* (%)	4 (16%)	16 (64%)	12 (48%)
Correct estimation of self-reported physical activity, *n* (%)	0	0	6 (24%)
Underestimation of self-reported physical activity, *n* (%)	21 (84%)	9 (36%)	7 (28%)

*Healthy controls*			
Overestimation of self-reported physical activity, *n* (%)	18 (34%)	25 (47%)	30 (57%)
Correct estimation of self-reported physical activity, *n* (%)	0	0	9 (17%)
Underestimation of self-reported physical activity, *n* (%)	35 (66%)	28 (53%)	14 (26%)

**Table 4:** Influencing factors on the overestimation of moderate physical activity.

*N* = 41	Beta	SE	95%-CI	*p*
*Model 1*				
Age (years)	3.584	2.390	[−1.263; 8.431]	0.142
Sex: “men” versus “women”	15.262	26.611	[−38.708; 69.232]	0.570
Body mass index (kg/m^2^)	2.514	3.370	[−4.320; 9.348]	0.460
Study group: “*healthy controls” *versus* “low back pain”*	76.427	79.996	[−85.813; 238.667]	0.346

*Model 2*				
Age (years)	1.102	1.089	[−1.117; 3.320]	0.319
Sex: “men” versus “women”	3.382	12.673	[−22.432; 29.196]	0.791
Body mass index (kg/m^2^)	−0.656	1.580	[−3.873; 2.562]	0.681
Study group: “*healthy controls” *versus* “low back pain”*	−1.436	36.858	[−76.512; 73.640]	0.969
Self-reported workplace physical activity (min/week)	0.072	0.007	[0.057; 0.087]	**<0.001** ^*∗*^
Self-reported leisure time physical activity (min/week)	0.060	0.010	[0.040; 0.081]	**<0.001** ^*∗*^
Self-reported transportation activity (min/week)	0.126	0.016	[0.093; 0.158]	**<0.001** ^*∗*^
Objective achievement of the WHO recommendations: “≥30 min physical activity/day” versus “<30 min physical activity/day”	−23.444	14.060	[−52.083; 5.196]	0.105

Dependent variable: overestimation of self-reported moderate physical activity (GPAQ/day-accelerometer/day); ^*∗*^statistically significant at the significance level *p* < 0.05; model 1: *R* ^2^ = 0.069; model 2: *R* ^2^ = 0.820.

**Table 5:** Influencing factors on the overestimation of vigorous physical activity.

*N* = 42	Beta	SE	95%-CI	*p*
*Model 1*				
Age (years)	0.540	2.190	[−3.899; 4.978]	0.807
Sex: “men” versus “women”	−28.289	22.918	[−74.726; 18.147]	0.225
Body mass index (kg/m^2^)	−1.329	2.163	[−5.711; 3.053]	0.543
Study group: “*healthy controls” *versus* “low back pain”*	−33.590	73.603	[−182.724; 115.544]	0.651

*Model 2*				
Age (years)	−0.527	1.137	[−2.843; 1.789]	0.646
Sex: “men” versus “women”	5.320	11.678	[−18.467; 29.107]	0.652
Body mass index (kg/m^2^)	−0.984	1.220	[−3.468; 1.501]	0.426
Study group: “*healthy controls” *versus* “low back pain”*	−8.524	38.764	[−87.484; 70.436]	0.827
Self-reported workplace physical activity (min/week)	0.059	0.007	[0.045; 0.073]	**<0.001** ^*∗*^
Self-reported leisure time physical activity (min/week)	0.077	0.009	[0.059; 0.096]	**<0.001** ^*∗*^
Self-reported transportation activity (min/week)	0.006	0.014	[−0.023; 0.035]	0.680
Objective achievement of the WHO recommendations: “≥30 min physical activity/day” versus “<30 min physical activity/day”	−5.114	13.645	[−32.909; 22.681]	0.710

Dependent variable: overestimation of self-reported vigorous physical activity (GPAQ/day-accelerometer/day); ^*∗*^statistically significant at the significance level *p* < 0.05; model 1: *R* ^2^ = 0.004; model 2: *R* ^2^ = 0.777.

**Table 6:** Influencing factors on the underestimation of sedentary time.

*N* = 56	Beta	SE	95%-CI	*p*
*Model 1*				
Age (years)	6.169	3.332	[−0.521; 12.858]	0.070
Sex: “men” versus “women”	−12.942	33.948	[−81.095; 55.211]	0.705
Body mass index (kg/m^2^)	5.597	4.158	[−2.751; 13.945]	0.184
Study group: “*healthy controls” *versus* “low back pain”*	375.692	110.393	[154.069; 597.316]	**0.001** ^*∗*^

*Model 2*				
Age (years)	6.697	3.505	[−0.354; 13.748]	0.062
Sex: “men” versus “women”	−15.665	36.393	[−88.878; 57.549]	0.669
Body mass index (kg/m^2^)	5.205	4.365	[−3.576; 13.987]	0.239
Study group: “*healthy controls” *versus* “low back pain”*	384.219	117.233	[148.377; 620.062]	**0.002** ^*∗*^
Self-reported workplace physical activity (min/week)	0.005	0.023	[−0.042; 0.052]	0.825
Self-reported leisure time physical activity (min/week)	−0.029	0.084	[−0.199; 0.140]	0.729
Self-reported transportation activity (min/week)	0.013	0.045	[−0.078; 0.104]	0.770
Objective achievement of the WHO recommendations: “≥30 min physical activity/day” versus “<30 min physical activity/day”	40.008	40.106	[−40.674; 120.691]	0.324

Dependent variable: underestimation of self-reported sedentary time (GPAQ/day-accelerometer/day); ^*∗*^statistically significant at the significance level *p* < 0.05; model 1: *R* ^2^ = 0.295; model 2: *R* ^2^ = 0.253.

## References

[1] US Department of Health and Human Services (1996). *Physical Activity and Health: A Report of the Surgeon General*.

[2] Lee I.-M., Shiroma E. J., Lobelo F. (2012). Effect of physical inactivity on major non-communicable diseases worldwide: an analysis of burden of disease and life expectancy. *The Lancet*.

[3] World Health Organization (WHO) (2011). *Global Status Report on Noncommunicable Diseases 2010*.

[4] Warburton D. E. R., Nicol C. W., Bredin S. S. D. (2006). Health benefits of physical activity: the evidence. *Canadian Medical Association Journal*.

[5] Olsson S. J. G., Ekblom Ö., Andersson E., Börjesson M., Kallings L. V. (2016). Categorical answer modes provide superior validity to open answers when asking for level of physical activity: a cross-sectional study. *Scandinavian Journal of Public Health*.

[6] Caspersen C. J., Powell K. E., Christenson G. (1985). Physical activity, exercise and physical fitness: definitions and distinctions for health-related research. *Public Health Reports*.

[7] Clark D. O. (2001). Issues of adherence, penetration, and measurement in physical activity effectiveness studies. *Medical Care*.

[8] Müller C., Winter C., Rosenbaum D. (2010). Aktuelle objektive Messverfahren zur Erfassung körperlicher Aktivität im Vergleich zu subjektiven Erhebungsmethoden. *DZSM*.

[9] Beneke R., Leithäuser R. M. (2008). Körperliche Aktivität im Kindesalter-Messverfahren. *DZSM*.

[10] Lee P. H., Macfarlane D. J., Lam T., Stewart S. M. (2011). Validity of the international physical activity questionnaire short form (IPAQ-SF): a systematic review. *International Journal of Behavioral Nutrition and Physical Activity*.

[11] Prince S. A., Adamo K. B., Hamel M. E., Hardt J., Connor Gorber S., Tremblay M. (2008). A comparison of direct versus self-report measures for assessing physical activity in adults: a systematic review. *The International Journal of Behavioral Nutrition and Physical Activity*.

[12] Grimm E. K., Swartz A. M., Hart T., Miller N. E., Strath S. J. (2012). Comparison of the IPAQ-short form and accelerometry predictions of physical activity in older adults. *Journal of Aging and Physical Activity*.

[13] Celis-Morales C. A., Perez-Bravo F., Ibañez L., Salas C., Bailey M. E. S., Gill J. M. R. (2012). Objective vs. self-reported physical activity and sedentary time: effects of measurement method on relationships with risk biomarkers. *PLoS ONE*.

[14] Lagersted-Olsen J., Korshøj M., Skotte J., Carneiro I. G., Sogaard K., Holtermann A. (2014). Comparison of objectively measured and self-reported time spent sitting. *International Journal of Sports Medicine*.

[15] van Weering M. G. H., Vollenbroek-Hutten M. M. R., Hermens H. J. (2011). The relationship between objectively and subjectively measured activity levels in people with chronic low back pain. *Clinical Rehabilitation*.

[16] Watkinson C., van Sluijs E. M. F., Sutton S., Hardeman W., Corder K., Griffin S. J. (2010). Overestimation of physical activity level is associated with lower BMI: a cross-sectional analysis. *The International Journal of Behavioral Nutrition and Physical Activity*.

[17] Schaller A., Froboese I. (2014). Movement coaching: study protocol of a randomized controlled trial evaluating effects on physical activity and participation in low back pain patients. *BMC Musculoskeletal Disorders*.

[18] Frick F., Sperlich B., Schaller A., Grieben C., Froböse I. (2013). BIBK—Bewegung ins Berufskolleg. Wie sieht eine nachhaltige bewegungsbezogene gesundheitsförderung im Berufskolleg aus?. *IMPULSE*.

[19] Armstrong T., Bull F. (2006). Development of the World Health Organization Global Physical Activity Questionnaire (GPAQ). *Journal of Public Health*.

[20] Bull F. C., Maslin T. S., Armstrong T. (2009). Global physical activity questionnaire (GPAQ): nine country reliability and validity study. *Journal of Physical Activity & Health*.

[21] Chu A. H. Y., Ng S. H. X., Koh D., Müller-Riemenschneider F., Brucki S. (2015). Reliability and validity of the self- and interviewer-administered versions of the Global Physical Activity Questionnaire (GPAQ). *PLoS ONE*.

[22] Herrmann S. D., Heumann K. J., Der Ananian C. A., Ainsworth B. E. (2013). Validity and reliability of the global physical activity questionnaire (GPAQ). *Measurement in Physical Education and Exercise Science*.

[23] Melanson E. L., Freedson P. S. (1995). Validity of the computer science and applications, Inc. (CSA) activity monitor. *Medicine and Science in Sports and Exercise*.

[24] Kelly L. A., McMillan D. G. E., Anderson A., Fippinger M., Fillerup G., Rider J. (2013). Validity of actigraphs uniaxial and triaxial accelerometers for assessment of physical activity in adults in laboratory conditions. *BMC Medical Physics*.

[25] Liu A.-L., Li Y.-P., Song J., Pan H., Han X.-M., Ma G.-S. (2005). Study on the validation of the computer science application's activity monitor in assessing the physical activity among adults using doubly labeled water method. *Zhonghua Liuxingbingxue Zazhi*.

[26] Freedson P. S., Melanson E., Sirard J. (1998). Calibration of the computer science and applications, Inc. accelerometer. *Medicine and Science in Sports and Exercise*.

[27] Troiano R. P. (2007). Large-scale applications of accelerometers: new frontiers and new questions. *Medicine and Science in Sports and Exercise*.

[28] World Health Organization (WHO) (2010). *Global Recommendations on Physical Activity for Health*.

[29] Bland J. M., Altman D. G. (1986). Statistical methods for assessing agreement between two methods of clinical measurement. *The Lancet*.

[30] Rudolf K., Schaller A., Frick F., Grieben C., Froböse I. (2015). *Measuring Physical Activity Awareness in Early Adulthood. A Cross-Sectional Study with Vocational Students in Germany*.

[31] van Sluijs E. M. F., Griffin S. J., van Poppel M. N. M. (2007). A cross-sectional study of awareness of physical activity: associations with personal, behavioral and psychosocial factors. *International Journal of Behavioral Nutrition and Physical Activity*.

[32] Visser M., Brychta R. J., Chen K. Y., Koster A. (2014). Self-reported adherence to the physical activity recommendation and determinants of misperception in older adults. *Journal of Aging and Physical Activity*.

[33] Canning K. L., Brown R. E., Jamnik V. K., Salmon A., Ardern C. I., Kuk J. L. (2014). Individuals underestimate moderate and vigorous intensity physical activity. *PLoS ONE*.

[34] Long G. H., Brage S., Wareham N. J. (2013). Socio-demographic and behavioural correlates of physical activity perception in individuals with recently diagnosed diabetes: results from a cross-sectional study. *BMC Public Health*.

[35] Godino J. G., Watkinson C., Corder K., Sutton S., Griffin S. J., Van Sluijs E. M. F. (2014). Awareness of physical activity in healthy middle-aged adults: a cross-sectional study of associations with sociodemographic, biological, behavioural, and psychological factors. *BMC Public Health*.

[36] Tudor-Locke C. E., Myers A. M. (2001). Challenges and opportunities for measuring physical activity in sedentary adults. *Sports Medicine*.

[37] Altschuler A., Picchi T., Nelson M., Rogers J. D., Hart J., Sternfeld B. (2009). Physical activity questionnaire comprehension: lessons from cognitive interviews. *Medicine and Science in Sports and Exercise*.

[38] Loney T., Standage M., Thompson D., Sebire S. J., Cumming S. (2011). Self-report vs. objectively assessed physical activity: which is right for public health?. *Journal of Physical Activity and Health*.

[39] Lechner L., Bolman C., van Dijke M. (2006). Factors related to misperception of physical activity in the Netherlands and implications for health promotion programmes. *Health Promotion International*.

[40] Ronda G., van Assema P., Brug J. (2001). Stages of change, psychological factors and awareness of physical activity levels in the Netherlands. *Health Promotion International*.

